# *Asparagus cochinchinensis* extract ameliorates menopausal depression in ovariectomized rats under chronic unpredictable mild stress

**DOI:** 10.1186/s12906-020-03121-0

**Published:** 2020-10-27

**Authors:** Hye Ryeong Kim, Young-Ju Lee, Tae-Wan Kim, Ri-Na Lim, Dae Youn Hwang, Jeffrey J. Moffat, Soonil Kim, Joung-Wook Seo, Minhan Ka

**Affiliations:** 1 Pharmacology and Drug Abuse Research Group, Research Center for Convergence Toxicology, Korea Institute of Toxicology, KRICT, Daejeon, 34114 Republic of Korea; 2 Department of Biomaterials Science, College of Natural Resources and Life Science/Life and Industry Convergence Research Institute, Pusan National University, Miryang, 50463 Republic of Korea; 3 Laboratory Animal Center, Korea Brain Research Institute, Daegu, 61062 Republic of Korea; 4 Department of Neurology, University of California, San Francisco, San Francisco, CA 94143 USA; 5 Olmanfood Co., Ltd, Seoul, 03709 Republic of Korea

**Keywords:** Menopausal depression, Ovariectomized rats, *Asparagus cochinchinensis*, Chronic mild stress, Inflammatory cytokines, Corticosterone, Depression-like behavior, BDNF-TrkB signaling

## Abstract

**Background:**

Depression is a serious and common psychiatric disorder generally affecting more women than men. A woman’s risk of developing depression increases steadily with age, and higher incidence is associated with the onset of menopause. Here we evaluated the antidepressant properties of *Asparagus cochinchinensis (*AC) extract and investigated its underlying mechanisms in a rat menopausal depression model.

**Methods:**

To model this menopausal depression, we induced a menopause-like state in rats via ovariectomy and exposed them to chronic unpredictable mild stress (CUMS) for 6 weeks, which promotes the development of depression-like symptoms. During the final 4 weeks of CUMS, rats were treated with either AC extract (1000 or 2000 mg/kg, PO), which has been reported to provide antidepressant effects, or with the tricyclic antidepressant imipramine (10 mg/kg, IP).

**Results:**

We report that CUMS promotes depression-like behavior and significantly increases serum corticosterone and inflammatory cytokine levels in the serum of ovariectomized (OVX) rats. We also found that CUMS decreases the expression of brain-derived neurotrophic factor (BDNF) and its primary receptor, tropomyosin receptor kinase B (TrkB), in OVX rats, and treatment with AC extract rescues both BDNF and TrkB expression levels.

**Conclusion:**

These results suggest that AC extract exerts antidepressant effects, possibly via modulation of the BDNF-TrkB pathway, in a rat model of menopausal depression.

**Supplementary Information:**

The online version contains supplementary material available at 10.1186/s12906-020-03121-0.

## Background

Depression is a highly prevalent neuropsychiatric disorder, which is more prevalent in women [1, 2]. Stages involving fluctuations or reductions in ovarian hormone levels, i.e. postpartum and menopausal phases, are accompanied by the highest risk of developing depression in adulthood [3, 4]. Menopausal depression typically occurs between the ages of 45 and 55 years old, and can contribute to serious, enduring physical and mental ailments [5, 6]. During menopause, the ovaries stop producing estrogen, which can result in multiple symptoms, including vasomotor symptoms, urogenital atrophy, osteoporosis, cardiovascular disease, cancer, psychiatric abnormalities, cognitive decline, and sexual problems [7, 8]. Ovariectomy is commonly used to model menopause in animals, as depletion of female gonadal hormones mimics estrogen depletion in menopausal women [9, 10]. Others have reported that OVX rodents exhibit increased anxiety- and depression-like behaviors, which show improvement with estrogen replacement [11, 12].

Chronic unpredictable mild stress (CUMS) is frequently used to elicit depression-like behavior in rodents [13, 14]. After 5–8 weeks of CUMS, rodents exhibit hyperactivity of the hypothalamic-pituitary adrenal (HPA) axis and corticosterone release, as well as anxiety- and depression-like behavioral phenotype [15, 16]. CUMS also directly reduces brain-derived neurotrophic factor (BDNF) expression and neurogenesis in the hippocampus [17, 18]. This reduction in hippocampal BDNF levels can be rescued via treatment with antidepressant drugs [19, 20]. BDNF binds selectively to the receptor tyrosine kinase, TrkB, which induces receptor phosphorylation. TrkB activation initiates several downstream signaling cascades, including the Ras/ERK, PLC-γ, and PI3K/AKT pathways [21, 22]. BDNF/TrkB signaling plays important roles in several aspects of brain development and function, such as neurogenesis, cell survival, neurite outgrowth, synaptogenesis, and learning and memory [23–25]. Upstream, PLC-γ directly induces a rise in intracellular Ca^2+^, thus activating the Ca^2+^/calmodulin dependent kinase (CaMKII) [26, 27]. CaMKII then activates BDNF expression in certain neurons via activation of the transcription factor CREB [28, 29].

AC extracts reportedly provide neuroprotective, anti-psychotic, and anti-depressant effects [30–32], as well as improved memory protection against amnesia in rodent models [33]. In addition, extracts from a related species, *Asparagus,* have been reported to possess anti-inflammatory properties [34, 35]. The primary functional biochemical unit of root extracts from the *Asparagus* family are steroidal saponins, called shatavrins [36, 37]. However, the mechanisms by which AC extract and/or shatavrins impart anti-depressant effects have not been completely explored.

In the current study we describe one mechanism by which AC extract relieves depression-like symptoms in CUMS-exposed OVX rats. CUMS significantly reduces BDNF-TrkB signaling in OVX rats, which results in depression -like behaviors and promotes the expression of pro-inflammatory cytokines and corticosterone. Treatment with AC extract ameliorates depression-like behavioral symptoms and restores BDNF and TrkB expression. These findings indicate the potential therapeutic efficacy of AC extract for treating menopausal depression and suggest a mechanism by which AC extract may relieve depression-like symptoms in rats.

## Methods

### Preparation of Asparagus cochinchinensis (AC) extract

The roots of *Asparagus cochinchinensis* were collected from the Henan Province, China in 2017 (Tianjin Pharmacn Medical Technology Co., Ltd.) and identified by oriental medical doctor. S. Kim (Olmanfood Co., Ltd., Seoul, Republic of Korea). Voucher specimens of the authenticated raw materials were deposited in the Olmanfood Company. Roots of *Asparagus cochinchinensis* were hot air-dried for 12 h at 60 °C. The dried roots were extracted with distilled water at 100 °C for 2 h [38, 39]. *Asparagus cochinchinensis* (AC) root extract was filtered and concentrated under reduced pressure at 60 °C for 12 h to obtain concentrated one. Finally, the AC extract was dried in a freezing drying for 72 h to obtain the AC extract stored in refrigerator at 4 °C for experimental usage.

### Animals and housing conditions

Five-week old female Wistar rats (100–120 g) were purchased from the Orient Bio Inc. (Seoul, Republic of Korea). The animals were housed 1 rat per cage under the following conditions: temperature (23 ± 3 °C), humidity (30–70%), with standard rodent chow and water available ad libitum. Rats were maintained on a 12 h light/dark cycle (lights on at 8:00 a.m.). Prior to the test procedure, rats were acclimatized to the laboratory for 2 weeks. All experimental procedures were approved by the Institutional Animal Care and Use Committee at the Korea Institute of Toxicology and met National Institutes of Health guidelines for the care and use of laboratory animals (KIT-IACUC; Approval Number 1804–0138 and 1806–0228).

### General procedures and groups

**Fig. 1:**
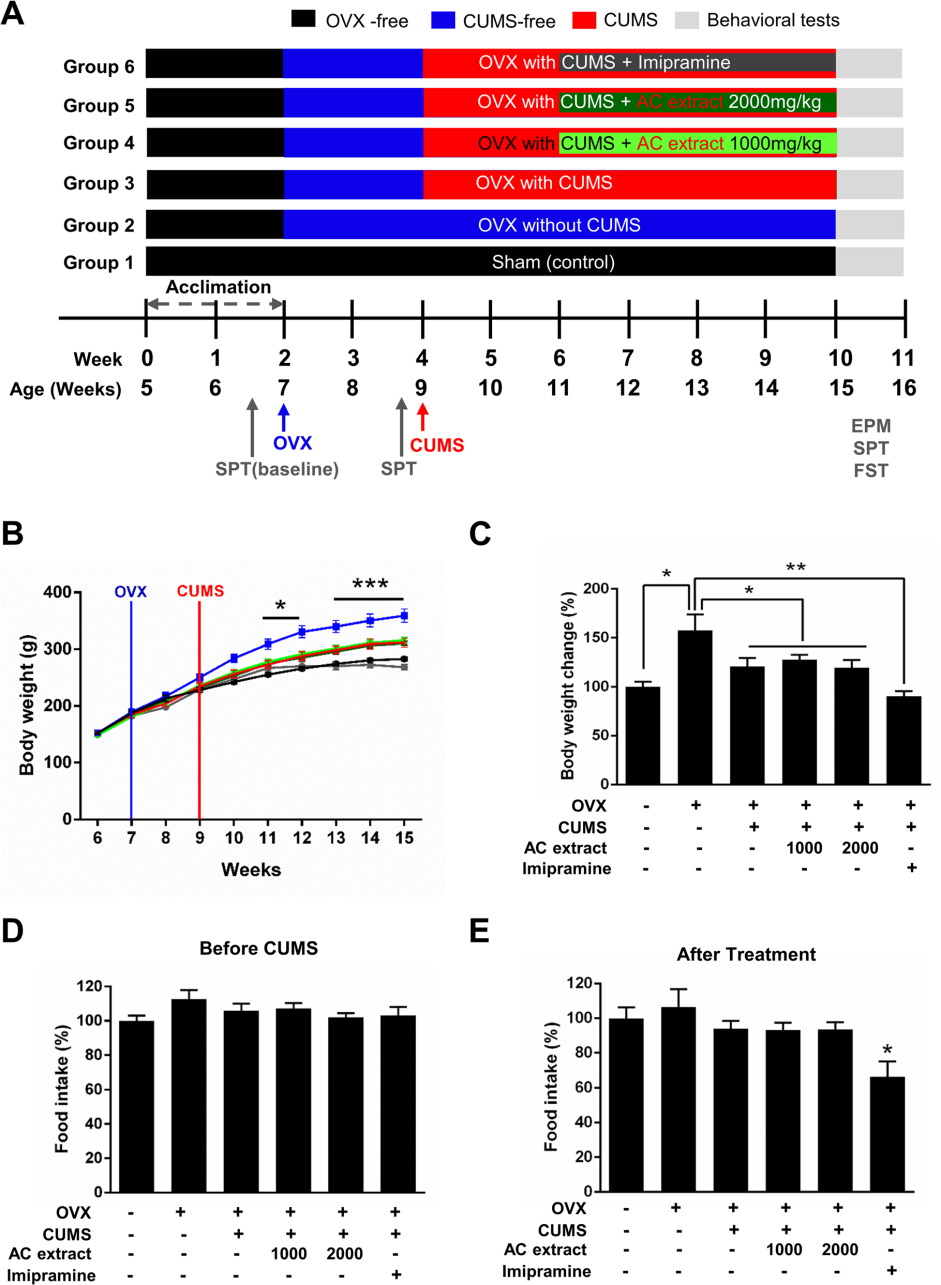
Effect of AC extract on body weight and food intake in OVX rats with CUMS exposure. **a** Schematic of the experimental design and group. Rats induce menopause by OVX. After recovery, the CUMS groups of animals received chronic unpredictable mild stress (CUMS) for 42 days. After 2 weeks of initial CUMS exposure, rats received AC extract or imipramine for 4 weeks. Subsequently, rats were evaluated depression-like behavior and biochemical analysis. **b** The effects of AC extract on body weight of rats. The body weight was measured once a week. *N* = 12 rats for each experimental group. Data represent mean ± SEM. Statistical significance was determined by multiple t-test with Bonferonni correction test. *P < 0.05, ***P < 0.001. **c** The ratio of in body weight gain (Δweight = Final body weight - Initial body weight). OVX increase body weight in rats, however, the body weight gain decreased in OVX rats after 6 weeks of CUMS exposure. *N* = 12 rats for each experimental group. Statistical significance was determined by multiple t-test with Bonferonni correction test. *P < 0.05, **P < 0.01. **d**, **e** The effects of AC extract on food intake of rats. The rate of food intake before CUMS exposure (D) and after CUMS exposure (E). *N* = 12 rats for each experimental group. Data represent mean ± SEM. Statistical significance was determined by multiple t-test with Bonferonni correction test. *P < 0.05

The female rats were housed for at least 2 weeks under controlled conditions before experiments began. 12 female rats were not ovariectomized and 60 female rats were ovariectomized bilaterally to remove the principal source of endogenous estrogen and allowed 2 weeks for recovery post-surgery before being used in experiments. The rats were divided into six groups and subjected to the following experimental conditions: sham (non OVX, non CUMS), OVX (OVX, non CUMS), OVX + CUMS (OVX, exposed to CUMS), OVX + CUMS + AC extract (1000 mg/kg), OVX + CUMS + AC extract (2000 mg/kg), and OVX + CUMS + imipramine (10 mg/kg). AC extract was administered orally to rats in two dosages, 1000 mg/kg or 2000 mg/kg, dissolved in sterile water. Control animals received sterile water only. Imipramine was administered via intraperitoneal injection. Body weight and food intake were recorded weekly throughout the experiment. Treatments began in the second week of CUMS and continued for four more weeks along with continued CUMS. At the conclusion of treatment, all rats were evaluated for anxiety- and depression-like behaviors using the sucrose preference test (SPT), elevated plus-maze (EPM), and forced swimming test (FST). Following behavioral tests, blood and tissues were collected for molecular and cellular studies. The general procedure is shown in Fig. 1a.

### Ovariectomy

Seven-week old female Wistar rats (200–220 g) were ovariectomized bilaterally to remove the principal source of endogenous estrogen. Briefly, rats were anesthetized with 4% isoflurane (HANA Pham co, Seoul, Republic of Korea) in 100% oxygen using an anesthetic hose. After disinfecting the skin (with alcohol and betadine), a 5.0 to 6.0 mm incision was made in the lower abdominal region to expose the uterus. Visible blood vessels were ligated, the ovaries removed, and the muscle layers and skin were sutured (Mersilk silk suture 4–0, Ethicon, Inc. NJ, USA). The animals were allowed to recover for 2 weeks after surgery.

### Chronic unpredictable mild stress (CUMS) exposure paradigms

All postoperative rats were separately housed and underwent the CUMS depression rat model. The CUMS strategy, first described by Banasr, was modified for this study [16, 40]. Briefly, out of 11 total stressors, two were used each day for a 6 week period in a random order: continuous overnight illumination (12 h), intermittent illumination (light on and off every 1 h; 3 h), paired cage (2–3 animals in each cage; 3 h), empty cage housing (18 h), physical restraint (2 h), 45° cage tilt (3 h), water deprivation (24 h), food deprivation (24 h), tail nip (1 min), white noise overnight (80–85 dB; 3 h), or wet bedding (400 mL water in 200 g sawdust bedding; 18 h). The sham group was housed in a different room where they had no contact with their stress exposed counterparts.

### Blood and brain tissue collection

At the end point of the experiment, rats were euthanized by excessive inhalation of isoflurane (Hana Pharm Co., Ltd. Seoul, Republic of Korea). Brains were surgically removed and were immediately frozen in liquid nitrogen and stored at − 80 °C until further analyses. Blood samples were obtained through cardiac puncture. Serum was then separated by centrifuging the blood at 2000 rpm for 20 min at 4 °C. Serum samples were stored at − 80 °C until further analysis.

### Sucrose preference test (SPT)

The sucrose preference test was performed as described previously [41] to assess anhedonia in rats. This test was carried out prior to the start of CUMS and at the end of each week. Rats were kept individually in separate cages and were allowed to adapt to two bottles of solution (filled with 1.0% sucrose solution) for 24 h. For the next 24 h, one bottle of sucrose solution was replaced with water. Then, the rats were subjected to 24 h of food and water deprivation, followed by exposure to two pre-weighed bottles of solution (1.0% sucrose solution and plain water, respectively) for 1 h. The position of the bottles was switched for each trial. After the test, the weight of sucrose solution and water consumed was recorded. Sucrose preference was calculated as a ratio of the weight of sucrose solution consumption to the weight of total fluid intake, sucrose preference = (sucrose intake / (sucrose intake + water intake)) × 100%.

### Forced swimming test (FST)

The forced swimming test was performed as described previously [42] to assess depression-like behavior in rats. A vertical glass cylinder (40 cm high, 30 cm in diameter) was filled with 25 ± 1 °C water to a depth of 30 cm. For testing, each rat was placed in the cylinder for 5 min, and duration of immobility, swimming, and climbing were scored. Water in the cylinder was changed for each rat. Immobility was recorded whenever animals stopped swimming and remained floating in the water, with their heads above the surface.

### Elevated plus maze test (EPM)

The elevated plus maze test was performed as described previously [43] to assess the anxiety-related behavior in rats. In the EPM test for rats, two opposite open arms (50 cm × 10 cm) and two opposite closed arms (50 cm × 10 cm × 40 cm) connected by a central square (10 × 10 cm) make up the apparatus, which is located 50 cm above the floor. The rats were individually placed in the central zone facing one of the open arms and a video camera mounted above the maze connected to a computer was used to monitor and score behavior during a 5-min experimental period. The percentage of open-arm entries (open/total entries × 100) and the proportion of time spent in the open arms (open/total time spent × 100) were calculated for each animal. Rats that fell down from the maze during testing were excluded from the study. Testing sessions were filmed using a digital camera (SLA-3580DN, Samsung Techwin, Seoul, Republic of Korea) and later analyzed with EthoVision XT software (Noldus Information Technology Inc. Leesburg, USA).

### Enzyme-linked immunosorbent assay (ELISA)

The serum and Brain tissue levels of CORT and cytokines including IL-1β, IL-6 and TNF-α, were measured using commercially available enzyme-linked immunosorbent assay (ELISA) kits (Enzo Life Sciences, Inc., Farmingdale, NY, USA; R&D Systems Inc., Minneapolis, MN, USA) according to the manufacturer’s instructions. Briefly, serial dilutions of protein standards and samples were added to 96-well ELISA plates, followed by biotinylated anti-IL-1β, IL-6 and TNF-α antibody. After rinsing with wash buffer, a prepared solution of an avidin–horseradish-peroxidase-conjugated complex was added, followed by incorporation of the substrate solution. The reaction was stopped using a stopping solution. The optical density was detected at 450 nm on a microplate reader (GloMax Discover Multimode Microplate Reader, Promega, Madison, WI, USA). The concentration of each sample was calculated from the linear equation derived from the standard curve of known concentrations of the cytokine.

### Western blotting

Western blotting was performed as described previously [44–46]. Tissue lysates from the hippocampal region were prepared using RIPA buffer and samples were centrifuged at 12,000 rpm for 10 min at 4 °C, then supernatants were collected and protein content was determined using the Pierce BCA Protein Assay Kit (Thermo Fisher Scientific, Waltham, MA, USA), following the manufacturer’s protocol. Proteins were separated on 8, 10%, or 15% SDS-PAGE gradient gels and transferred onto PVDF transfer membranes (Thermo Fisher Scientific, Waltham, MA, USA). Membranes were then incubated with rabbit anti-BDNF (ab108319, Abcam, Cambridge, UK), rabbit anti-TrkB (ab18987, Abcam, Cambridge, UK), rabbit anti-PSD95 (ab18258, Abcam, Cambridge, UK), rabbit anti-Synaptophysin (ab32594, Abcam, Cambridge, UK), rabbit anti-Akt (#9272, Cell Signalling Technology, Danvers, MA, USA), rabbit anti-p-Akt (#9271, Cell Signalling Technology, Danvers, MA, USA), rabbit anti-Erk1/2 (#4695, Cell Signalling Technology, Danvers, MA, USA), rabbit anti-p-Erk1/2 (#4377, Cell Signalling Technology, Danvers, MA, USA), mouse anti-CREB (#9104, Cell Signalling Technology, Danvers, MA, USA), or rabbit anti-p-CREB (#9198, Cell Signalling Technology, Danvers, MA, USA) and mouse anti-β-actin (A5316, Thermo Fisher Scientific, Waltham, MA, USA) at 4 °C overnight. Appropriate secondary antibodies conjugated to HRP (Thermo Fisher Scientific, Waltham, MA, USA) and the ECL reagents (Thermo Fisher Scientific, Waltham, MA, USA) were used for immunodetection.

For quantification of band intensity, blots from 3 independent experiments for each molecule of interest were used. Signals were measured using ImageJ software and represented by relative intensity versus control. β-actin was used as an internal control to normalize band intensity.

### Immunohistochemistry

Immunostaining of brain sections or dissociated cells was performed as described previously [47–49]. The following primary antibodies were used: rat anti-GFAP (#13–0300, Thermo Fisher Scientific, Waltham, MA, USA), rabbit anti-Iba1 (019–19,741, FUJIFILM Wako Pure Ceemical Corporation, Tokyo, Japen) antibody. Appropriate secondary antibodies conjugated with Alexa Fluor dyes (Thermo Fisher Scientific, Waltham, MA, USA) were used to detect primary antibodies.

### Statistical analysis

Normal distribution was tested using the Kolmogorov–Smirnov test, and variance was compared. Unless otherwise stated, statistical significance was determined by one-way or two-way analysis of variance (ANOVA) followed by the Bonferroni post hoc test for multiple comparisons. Data were analyzed using GraphPad Prism (GraphPad Software, Inc. La Jolla, CA, USA) and presented as mean (±) SEM. *P* values were indicated in figure legends.

## Results

### AC extract does not alter food consumption or body weight in OVX rats exposed to CUMS

To assess the efficacy of AC extract in treating menopausal depression, we first induced a menopause-like state by ovariectomizing a cohort of female rats. A control received a sham operation. Following recovery from surgery, we subjected OVX rats to 6 weeks of CUMS, to better model menopausal depression-like behavior. One control group received no OVX surgery or CUMS exposure and a second control group did not undergo CUMS following ovariectomy. After 2 weeks of initial CUMS exposure, the remaining ovariectomized animals received daily administration of AC extract (1000 mg/kg, 2000 mg/kg, PO) or imipramine (IMI) (10 mg/kg, IP) during an additional 4 weeks of CUMS (Fig. 1a).

OVX leads to marked increases in body weight in rats [12], while depression is often accompanied by weight loss [50]. We observed no significant difference in baseline body weights between the six experimental groups, but starting 3 weeks following surgery, OVX rats exhibited higher body weights than sham-operated controls. This increase in body weight in OVX rats persisted for the remainder of the 10 week study. CUMS, however, had no effect on the body weight of OVX rats (Fig. 1b). OVX rats undergoing CUMS and continuously treated with imipramine (10 mg/kg) exhibited lower body weights than controls, while treatment with AC extract had no significant effect on body weight (Fig. 1b). OVX rats not exposed to CUMS demonstrated a 57% increase in body weight over the course of the study, compared to sham-operated controls, while CUMS-exposed OVX rats did not exhibit significant weight gain during the experiment (Fig. 1c). We likewise did not observe any changes in the rate of food consumption in all six groups (Fig. 1d). Treatment with AC extract also had no impact on food intake in CUMS-exposed OVX rats, while imipramine injections significantly reduced consumption (Fig. 1e). These results confirm that OVX leads to significant weight gain in rats, while CUMS negatively regulates weight gain in OVX rats. Furthermore, AC extract has no effect on weight gain/loss or food consumption in OVX rats with or without CUMS, while imipramine promotes weight loss and reduces food consumption.

### AC extract and depression-like behavior in OVX rats exposed to CUMS

**Fig. 2:**
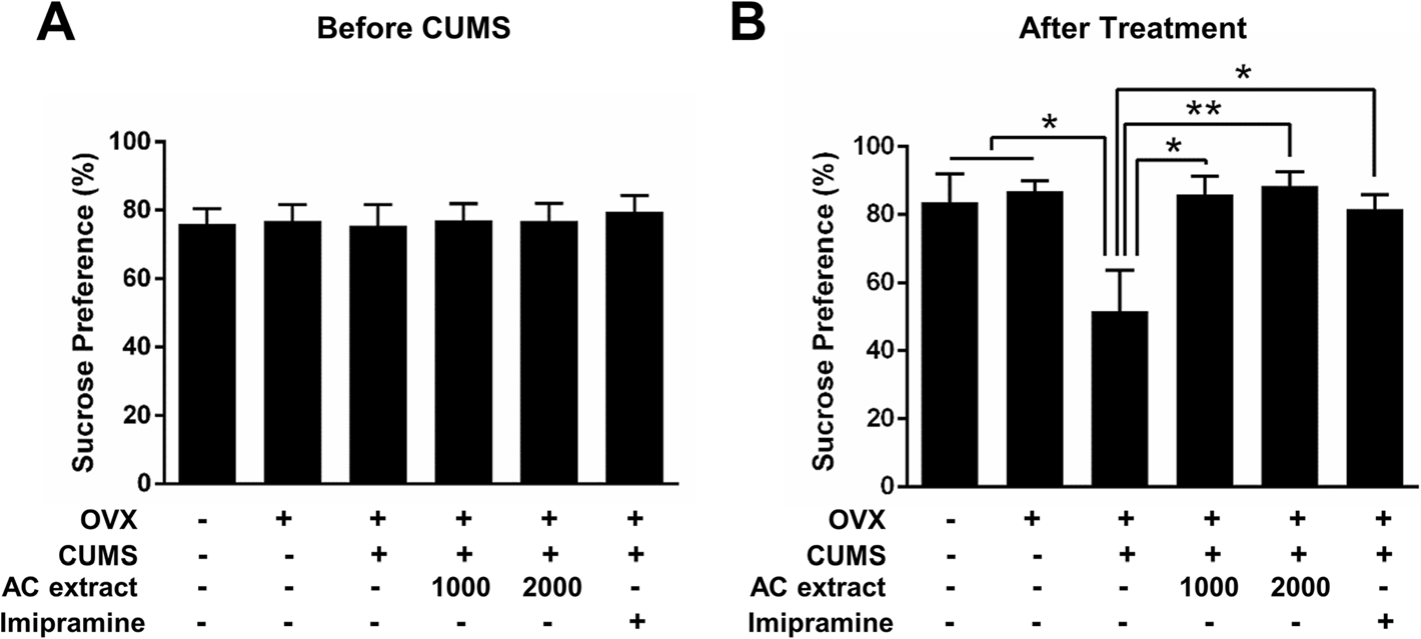
Effect of AC extract on sucrose intake in OVX rats with CUMS exposure. **a**, **b** The anhedonic behavior of rats during SPT. The rate of sucrose intake before CUMS exposure (**a**) and after CUMS exposure (**b**). AC extract administration restored CUMS-induced anhedonic behavior in the OVX rats. *N* = 12 rats for each experimental group. Data represent mean ± SEM. Statistical significance was determined by multiple t-test with Bonferonni correction test. *P < 0.05, **P < 0.01

To measure the efficacy of AC extract in treating menopausal depression-like behavior, we first assessed the hedonic state of OVX rats before and after CUMS exposure with the sucrose preference test (SPT). We observed no differences in sucrose preference in any of the six experimental groups at baseline (Fig. 2a). CUMS led to a 40% reduction in sucrose preference, indicative of depression-like behavior (Fig. 2b). Treatment with AC extract or imipramine successfully rescued sucrose preference to control levels, suggesting that AC extract is an effective treatment for depression-like behavior in OVX rats exposed to CUMS.

**Fig. 3:**
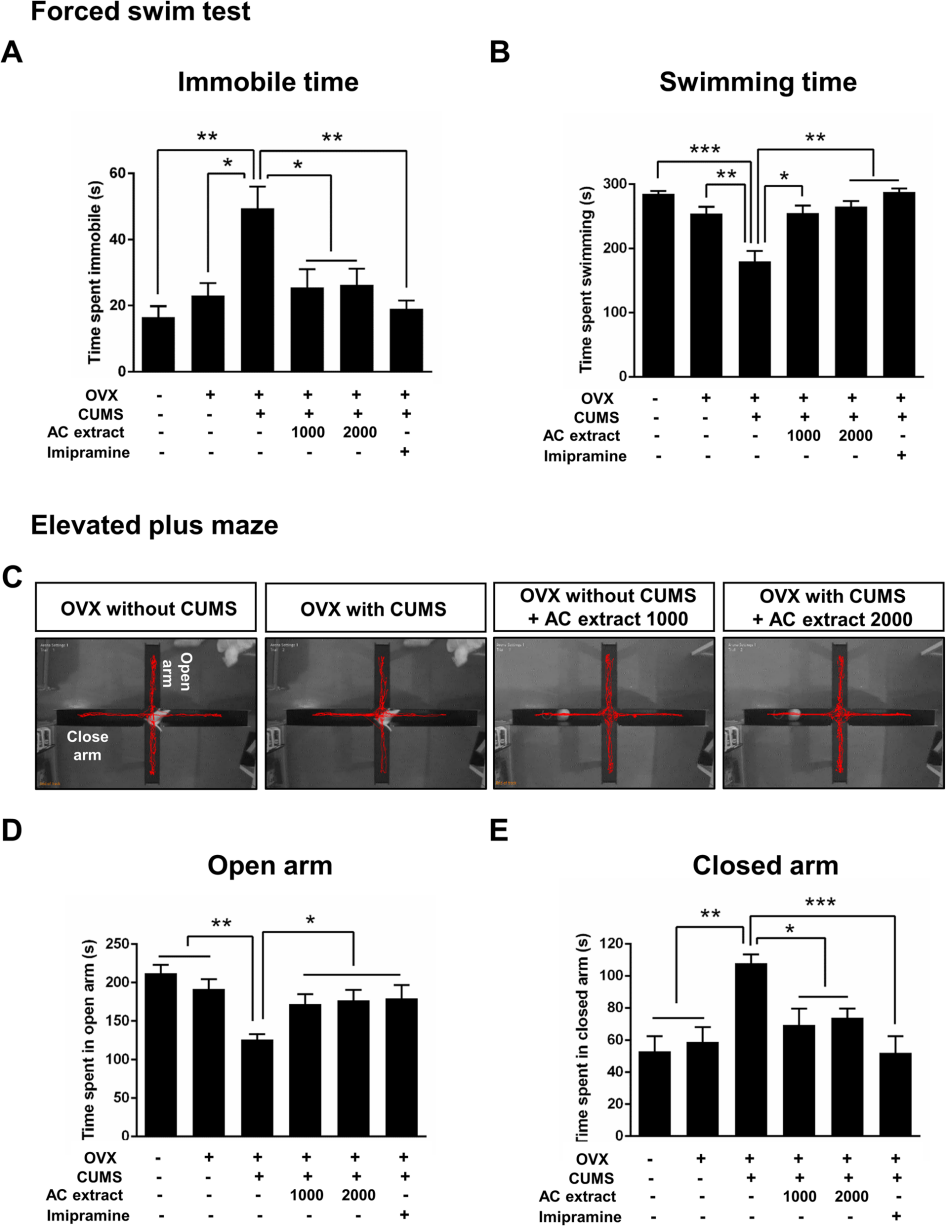
Effect of AC extract on depression-like behaviors in OVX rats with CUMS exposure. **a, b** The depression-like behavior of rats during FST. The time of immobility (**b**) and the time of swimming (**b**). AC extract administration decreased immobility times and increased swimming times in OVX rats with CUMS exposure. *N* = 12 rats for each experimental group. Data represent mean ± SEM. Statistical significance was determined by multiple t-test with Bonferonni correction test. *P < 0.05, **P < 0.01, ***P < 0.001. **c** The depression-like behavior of rats during EPM. **d**, **e** Quantification of (**c**). The spend time of open arm (**d**) and the spend time of closed arm (**e**). AC extract administration increased open arm spent times and decreased close arm spent times in OVX rats with CUMS exposure. *N* = 12 rats for each experimental group. Data represent mean ± SEM. Statistical significance was determined by multiple t-test with Bonferonni correction test. *P < 0.05, **P < 0.01, ***P < 0.001

To confirm the anti-depressant effects of AC extract observed above, we analyzed the immobility and swimming time of OVX rats exposed to CUMS following treatment with AC extract or imipramine. CUMS led to a significant increase in immobility time, and a corresponding decrease in swimming time, in OVX rats. Treatment with either AC extract or imipramine reversed this depression-like phenotype to normal levels (Fig. 3a-b). Overall, AC extract appears to be as effective as imipramine in treating menopausal depression in a rat model.

We next investigated the effects of AC extract on anxiety-like behavior in OVX rats undergoing CUMS. In the elevated plus maze (EPM), CUMS led to a significant decrease in open arm time in OVX rats, and a consequent increase in closed arm time (Fig. 3d-e), suggesting that CUMS incites anxiety-like behavior in OVX animals [51]. AC extract or imipramine treatment effectively returned open and closed arm times to baseline levels (Fig. 3d-e), indicating that AC extract may have anxiolytic, in addition to antidepressant, properties in OVX rats exposed to CUMS.

### AC extract reduces serum corticosterone and cytokine levels in OVX rats with CUMS exposure

**Fig. 4:**
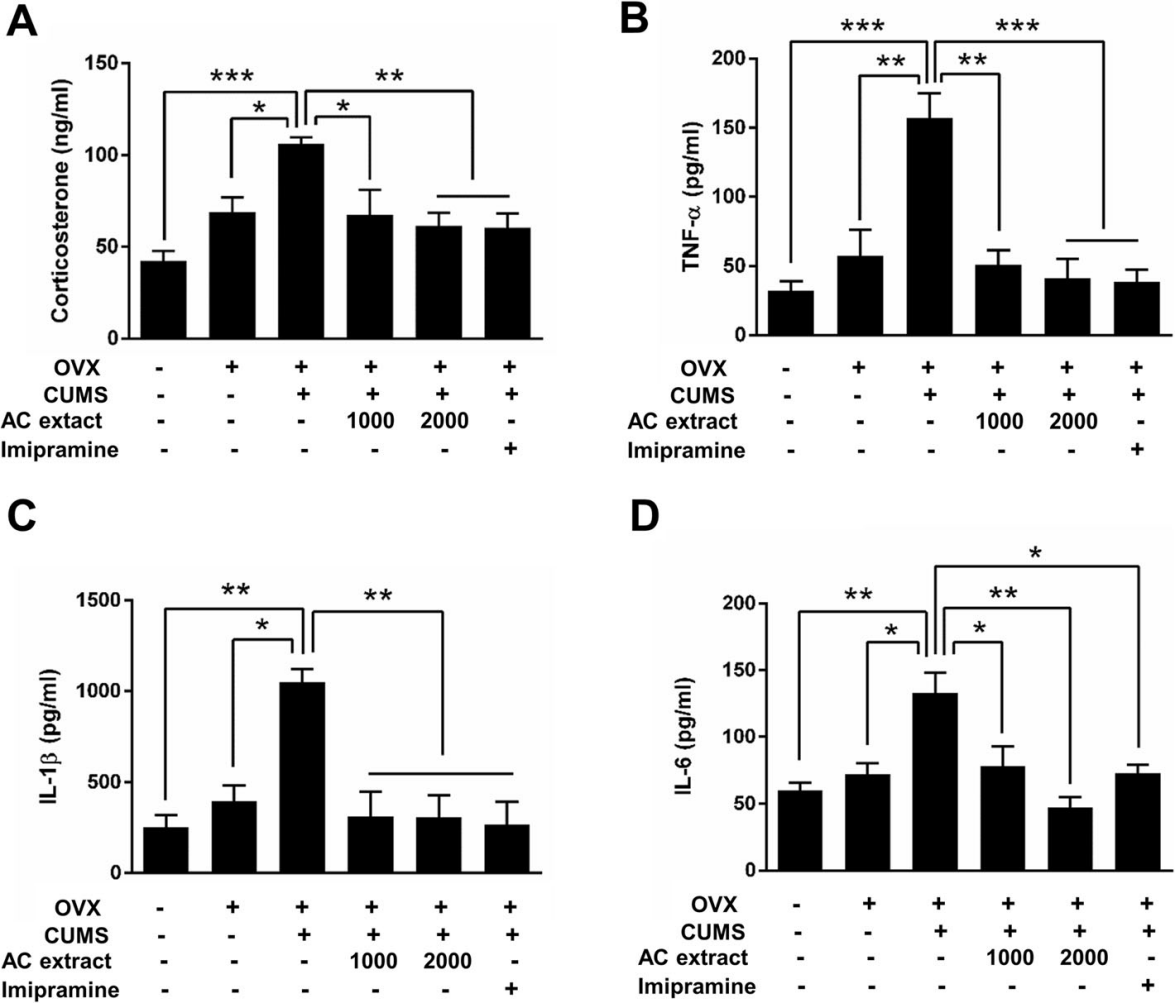
Effect of AC extract on serum corticosterone and pro-cytokines levels in OVX rats with CUMS exposure. **a** The level of serum corticosterone in rats was measured after AC extract treatment. AC extract administration suppressed serum corticosterone level in OVX rats with CUMS exposure. *N* = 5 independent ELISA from 5 rats for each experimental group. Data represent mean ± SEM. Statistical significance was determined by multiple t-test with Bonferonni correction test. *P < 0.05, **P < 0.01, ***P < 0.001. **b**, **c**, **d** The levels of serum pro-cytokines in rats were measured after AC extract treatment. AC extract administration suppressed serum TNF-α, IL-1β, IL-6 levels in OVX rats with CUMS exposure. *N* = 5 independent ELISA from 5 rats for each experimental group. Data represent mean ± SEM. Statistical significance was determined by multiple t-test with Bonferonni correction test. *P < 0.05, **P < 0.01, ***P < 0.001

Stress increases serum corticosterone levels [52]. As AC extract reduces stress-induced depression and anxiety-like behavior in OVX rats, we hypothesized that AC extract would reduce serum corticosterone levels as well. We report that CUMS increased serum corticosterone levels by 54% in OVX rats and that treatment with AC extract and or imipramine restored the corticosterone levels to normal ranges (Fig. 4a).

In addition to elevating serum corticosterone levels, stress can induce peripheral and central inflammation, neural damage and neuronal apoptosis [53]. We therefore examined the effects of AC extract on reducing circulating levels of several pro-inflammatory cytokines using ELISA. We found that CUMS significantly increased serum levels of TNF-α, IL-1β and IL-6 in OVX rats, but treatment with either AC extract or imipramine restores the levels of all of these pro-inflammatory cytokines to normal levels (Fig. 4b-d). Taken together, our results demonstrate that treatment with AC extract reduces corticosterone and pro-inflammatory cytokine levels in the serum of OVX rats exposed to CUMS.

### AC extract promotes hippocampal BDNF-TrkB signaling in OVX rats exposed to CUMS

**Fig. 5:**
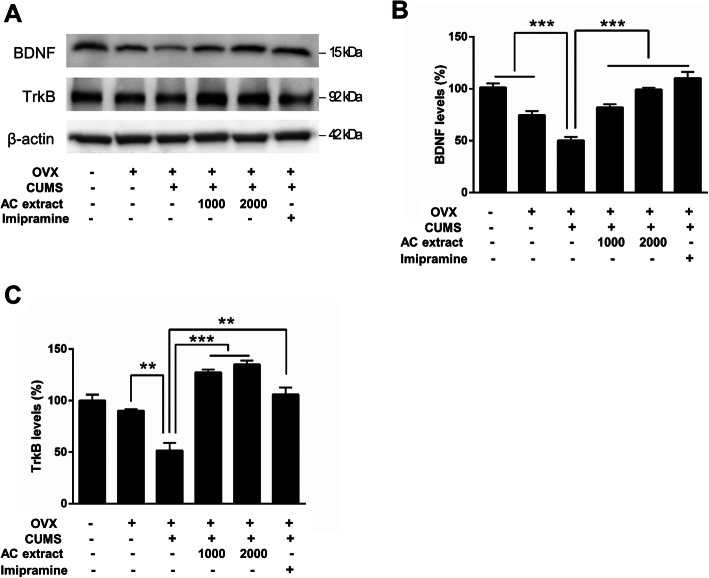
Effect of AC extract on BDNF and TrkB expression in OVX rats with CUMS exposure. **a** The levels of BDNF and TrkB expression in hippocampus of rats were measured by western blotting. AC extract administration restored BDNF and TrkB expression in OVX rats with CUMS exposure. Western blots show expression levels of BDNF and TrkB in hippocampus of rats. **b**, **c** Quantification of proteins shown in (**a**). The relative levels of the proteins were normalized to β-actin expression. The band intensities were measured using Image J. *N* = 3 independent ELISA from 5 rats for each experimental group. Data represent mean ± SEM. Statistical significance was determined by multiple t-test with Bonferonni correction test. **P < 0.01, ***P < 0.001

**Fig. 6:**
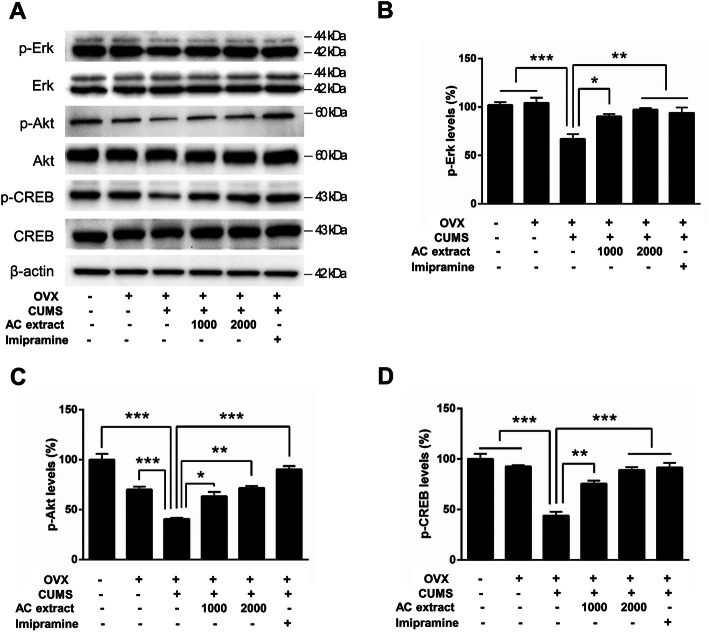
Effect of AC extract on kinases activity of BDNF downstream in OVX rats with CUMS exposure. **a** The levels of phospho-Erk, phospho-Akt, and phospho-CREB in hippocampus of rats were measured after AC extract treatment. AC extract administration restored Erk, Akt, and CREB phosphorylation in OVX rats with CUMS exposure. Western blots show phosphorylation levels of Erk, Akt, and CREB in hippocampus of rats. **b**, **c**, **d** Quantification of phospho-proteins shown in (**a**). The relative levels of phospho-proteins were normalized to total protein levels. The band intensities were measured using Image J. *N* = 3 independent ELISA from 5 rats for each experimental group. Data represent mean ± SEM. Statistical significance was determined by multiple t-test with Bonferonni correction test. *P < 0.05, **P < 0.01, ***P < 0.001

Chronic stress and depression are associated with reduced BDNF synthesis and decreased TrkB signaling in the hippocampus and cerebral cortex [54, 55]. With this in mind, we examined whether AC extract had an effect on BDNF and TrkB protein expression levels in the hippocampus of OVX rats. We found that CUMS significantly reduced BDNF and TrkB protein levels using western blot analysis, but that this reduction was erased when CUMS was accompanied by treatment with AC extract or imipramine (Fig. 5a-c). These results suggest that AC extract may have a role in regulating BDNF/TrkB signaling in the brain. Therefore, we next examined downstream BDNF/TrkB signaling in OVX rats by measuring phosphorylation of Erk and Akt kinases, as well as the transcription factor CREB, in hippocampal lysates. CUMS exposure effectively lowered phosphorylation of all three markers of downstream BDNF/TrkB signaling, but treatment with AC extract or imipramine restored phosphorylation levels of Erk, Akt and CREB to normal levels (Fig. 6a-d). Altogether, these data suggest that AC extract promotes BDNF and TrkB protein expression and downstream signaling in a rat model of menopausal depression. This indicates a potential mechanism for the anxiolytic and antidepressant actions of AC extract.

### AC extract restores synaptic marker expression level decreases due to CUMS in OVX rats

**Fig. 7:**
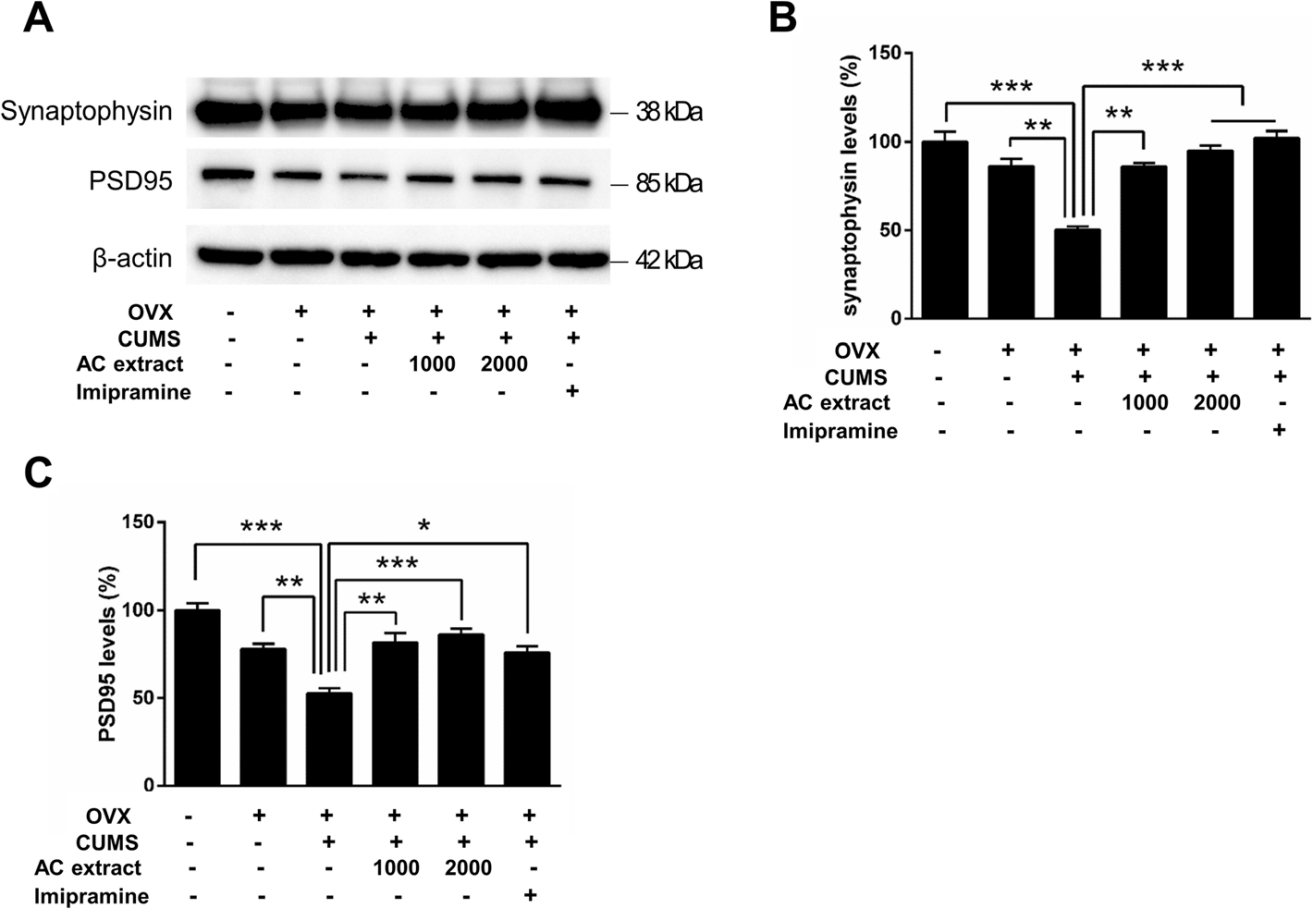
Effect of AC extract on synaptic markers expression in OVX rats with CUMS exposure. **a** The levels of Synaptophysin and PSD95 expression in hippocampus of rats were measured by western blotting. AC extract administration restored Synaptophysin and PSD95 expression in OVX rats with CUMS exposure. Western blots show expression levels of Synaptophysin and PSD95 in hippocampus of rats. **b**, **c** Quantification of proteins shown in (**a**). The relative levels of the proteins were normalized to β-actin expression. The band intensities were measured using Image J. *N* = 3 independent ELISA from 5 rats for each experimental group. Data represent mean ± SEM. Statistical significance was determined by multiple t-test with Bonferonni correction test. *P < 0.05, **P < 0.01, ***P < 0.001

BDNF is an important factor in hippocampal synaptic plasticity [56, 57] and BDNF deficits are directly implicated in clinical depression [58]. Since AC extract regulates BDNF expression and signaling, we next examined the effects of AC extract on stress-induced synaptic changes in the hippocampus. Using western blot analysis, we measured the expression of pre- and postsynaptic excitatory synaptic markers, synaptophysin and PSD95, respectively, in all six experimental groups. We found that CUMS significantly reduced synaptophysin and PSD95 protein levels in hippocampal lysates, but treatment with either imipramine or AC extract rescued excitatory synaptic marker expression to baseline levels (Fig. 7a-c). This suggests that AC extract positively regulates hippocampal excitatory synapses, presumably via BDNF/TrkB signaling.

### Treatment with AC extract rescues the reduction in hippocampal astrocyte density following CUMS

**Fig. 8:**
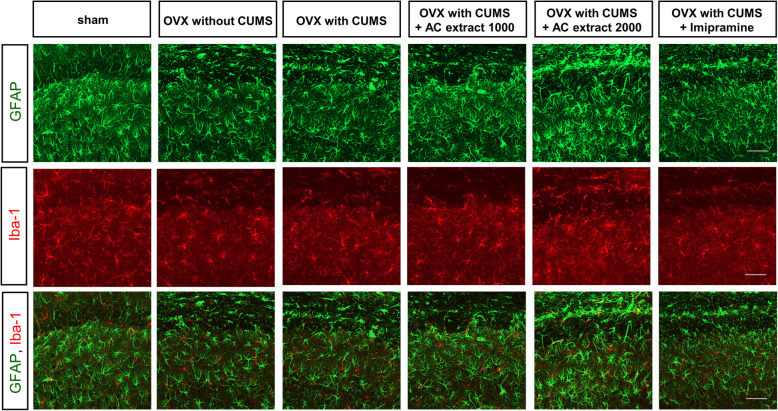
Effect of AC extract on astrocyte activity in OVX rats with CUMS exposure. Representative images show GFAP (astrocytic marker) and Iba-1 (microglial marker) staining in hippocampal region of rats. AC extract administration restored astrocyte activity in OVX rats with CUMS exposure. Scale bar, 50 μm

A reduction in GFAP-positive astrocytes in the hippocampus and prefrontal cortex is associated with major depressive disorder [59, 60]. We examined astrocyte and microglia density in the CA1 region of the hippocampus of rats from all 6 of our experimental groups, to assess the effects of CUMS and AC extract treatment on glia in the hippocampus. Immunohistochemical analysis revealed that the density of GFAP-positive astrocytes, but not Iba-1-positive microglia, was reduced in the CA1 region (Fig. 8). Interestingly, treatment with either imipramine or AC extract rescued CA1 astrocyte density to normal levels in OVX rats exposed to CUMS. These findings indicate that CUMS can reduce astrocyte density in the hippocampus of OVX rats, but that treatment with AC extract is sufficient to reverse this phenomenon.

## Discussion

In this study we induced a menopause-like state in rats via OVX and subjected them to CUMS to develop a model of menopausal depression. We report that these OVX rats that underwent CUMS exhibit depression- and anxiety-like behaviors and several cellular and molecular hallmarks of depression, and that treatment with AC extract ameliorates these symptoms. Species of *Asparagus* have been widely used in traditional Chinese medicine due to putative neuroprotective, antipsychotic and antidepressant effects. Here we evaluated the antidepressant properties of AC extract and investigated its underlying mechanisms in a rat menopausal depression model.

We found that OVX leads to significant weight gain in the absence of increased food intake, which aligns with potential menopause symptoms in humans, as estrogen and its receptors are essential components of the central network controlling food intake and energy metabolism [8]. Correspondingly, estrogen deficits have been shown to cause basal metabolism deficits and obesity [61]. The CUMS procedure utilized in this study, which consists of repeated exposure to varying, unpredictable, mild stressors [62], has been shown to reduce weight gain [63], or even to weight loss [16], in some rodent models. We report that OVX rats exposed to CUMS experience reduced weight gain, but treatment with AC extract has no additional effect on body weight in these animals. This implies that the changes in body weight due to menopause and may not be directly related to menopausal depression, as treatment with antidepressant compounds did not restore weight changes to normal levels.

Anhedonia is a key symptom of major depression [64] and the SPT is a common approach for assessing anhedonia-like states in animals [41]. Here we show that CUMS reduces the sucrose preference index in OVX rats, but administration of AC extract significantly increases sucrose intake back to baseline levels. Despair is the other main symptom of depression and the FST is thought to model the despair aspect of depression-like behavior in rodents [65]. We report that OVX rats exposed to CUMS also present with despair-like behavior in the FST and this phenotype is likewise reversed with AC extract treatment. In addition, we find that AC extract possesses anxiolytic properties, as treatment with AC extract reduces the anxiety-like behavior we observed in OVX rats exposed to CUMS. Our findings suggest that AC extract acts anti-anxiety and anti-depressive effect in the OVX rats with CUMS exposure. Overall, we show that AC extract is an effective treatment for the behavioral aspects of menopausal depression.

Elevated serum levels of cortisol are reported in patients diagnosed with depression [66]. Moreover, CUMS induces the accumulation of plasma corticosterone via dysfunction of the hypothalamic-pituitary-adrenal (HPA) axis [67]. Here we describe that CUMS also leads to increases in serum corticosterone levels in OVX rats, and that treatment with AC extract significantly reduces corticosterone to normal levels. Rodent CUMS exposure has also been reported to increase the levels of pro-inflammatory cytokines in both blood plasma and limbic brain regions [68], as well as infiltration of another pro-inflammatory cytokine, IL-6, into the brain [69]. We find that CUMS also increases the levels of all three of these pro-inflammatory cytokines in the serum of OVX rats, while AC extract administration significantly reduces pro-inflammatory cytokine levels. These findings provide molecular evidence for the antidepressant properties of AC extract, though it remains unclear whether AC extract is anti-inflammatory on its own, or if this reduction in serum pro-inflammatory cytokine levels is corollary to AC extracts primary mechanism.

Stress, which is a major contributor to depression, reduces BDNF expression and serum BDNF levels are lower in depressed patients [70, 71]. Chronic antidepressant treatment increases BDNF mRNA expression in the rat hippocampus. Furthermore, BDNF/TrkB signaling plays a key role in the pathophysiology of depression and has been found to be a therapeutic target for antidepressant drugs [72]. CUMS exposure in OVX rats leads to a reduction in BDNF and TrkB expression levels in the hippocampus and an accompanying decrease in downstream signaling, as evidenced by decreased phosphorylation of Erk, Akt and CREB [20, 28, 73]. Treatment with AC extract during CUMS, however, significantly increased both the expression levels of BDNF and TrkB proteins, as well as downstream signaling in the hippocampus. These findings provide a potential mechanistic explanation for the antidepressant effects of AC extract, suggesting that it activates BDNF/TrkB signaling in the hippocampus by promoting BDNF and TrkB protein expression. Further studies will be needed to confirm that the antidepressant effects of AC extract are indeed BDNF/TrkB-dependent.

Synaptic plasticity is important for normal brain function and the mechanisms underlying these changes have been linked to the pathophysiology and treatment of multiple neurobiological disorders, including depression [74]. Chronic antidepressant treatment can increase synaptic plasticity at several levels, including increased neurogenesis in the adult hippocampus, increased neurotrophic factor expression, and regulation of synapse formation [75, 76]. In patients with depression, impaired excitatory synaptic transmission leads to reduced activity in the mesolimbic reward circuitry [77, 78]. A previous study found that levels of the excitatory presynaptic membrane protein synaptophysin are reduced in frontal cortex samples from patients with bipolar disorder [79]. Similarly, expression levels of excitatory postsynaptic markers such as PSD95, NR2A, and NR2B are all reduced in the prefrontal cortex of major depression disorder patients [80]. We show here that CUMS exposure reduces the levels of synaptophysin and PSD95 in OVX rats, but AC extract administration is sufficient to restore the expression levels of both excitatory pre- and postsynaptic markers. Our findings suggest that AC extract may enhance excitatory synaptic plasticity in the hippocampus, which could in turn be involved in the antidepressant effects of AC extract in our rat model of menopausal depression.

Stress-based models of depression are associated with a reduction in GFAP expression [81]. GFAP fluorescence is significantly reduced in the hippocampal CA1 and CA2 regions in patients with depression [82]. Moreover, in both animals and humans, a reduction in astrocyte density is associated with neuropsychological disorders, such as memory impairments and depression [83]. We find that CUMS reduces the number of GFAP-positive astrocytes in the CA1 region of the hippocampus in OVX rats, however treatment with AC extract significantly rescues this decrease in GFAP-positive astrocytes. This finding provides another line of evidence for the potential use of AC extract to treat depression.

## Conclusions

We conclude from the present study that extract from the *Asparagus cochinchinensis* root ameliorates depression-like symptoms in a rat model of menopausal depression. AC extract rescues depression-like behaviors, maintains normal serum levels of pro-inflammatory cytokines, promotes excitatory synaptic plasticity and restores astrocyte density in the hippocampus. The rescue of these depression-like behaviors and depression-related cellular/molecular changes may be due to changes in BDNF/TrkB expression and signaling – which are downregulated in OVX rats exposed to CUMS but restored to pre-stress levels with treatment with AC extract or a traditional antidepressant. This study in an animal model provides insight into the pathophysiology of menopausal depression and suggests that AC extract contains compounds that could be useful in treating menopause-associated mood disorders.

## Supplementary Information

**Additional file 1.**

## Data Availability

The supporting materials can be obtained upon request via email to the corresponding author.

## Abbreviations

CUMS: Chronic unpredictable mild stress
AC: *Asparagus cochinchinensis*
OVX: *Ovariectomized*
*BDNF*: *Brain-derived neurotrophic factor*
*TrkB*: *Tropomyosin receptor kinase B*
*Ras*: *Small GTP-binding protein*
*ERK*: Extracellular-signal-regulated kinase
PLC-γ: Phosphoinositide phospholipase C
PI3K: Phosphatidylinositol 3-kinases
AKT: Serine/threonine kinase
CaMKII: Ca^2+^/calmodulin dependent kinase
SPT: Sucrose preference test
EPM: Elevated plus-maze
FST: Forced swimming test
SP: Sucrose intake
ELISA: Enzyme-linked immunosorbent assay
*CORT*: Corticosterone
IL-1β: Interleukin 1 beta
IL-6: Interleukin 6
TNF-α: Tumor necrosis factor alpha
PSD95: Postsynaptic density protein 95
CREB: cAMP response element-binding protein
GFAP: Glial fibrillary acidic protein
PO: Peroral
IP: Intraperitoneal
NR2A: Glutamate [NMDA] receptor subunit epsilon-1
NR2B: Glutamate [NMDA] receptor subunit epsilon-2
