# Supplementary material for: Asparagus cochinchinensis extract ameliorates menopausal depression in ovariectomized rats under chronic unpredictable mild stress
Source: BMC Complement Med Ther. 2020 Oct 27;20:325. doi: 10.1186/s12906-020-03121-0 (PMC7590795; doi:10.1186/s12906-020-03121-0)
Supplement: Supplementary file 1 — Additional file 1. [file 12906_2020_3121_MOESM1_ESM.pptx]

## Slide 1
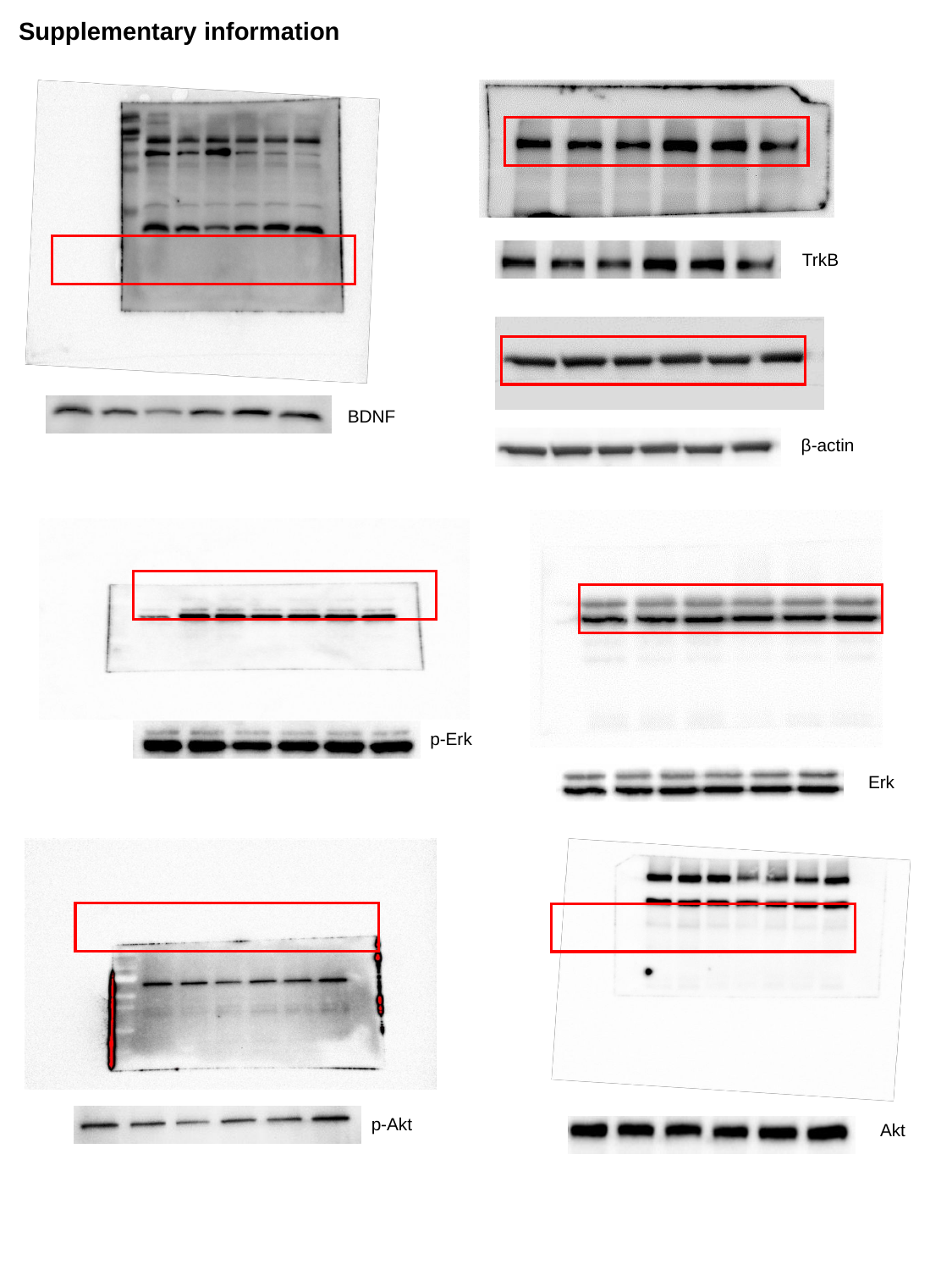

Supplementary information
TrkB
BDNF
β-actin
p-Erk
Erk
p-Akt
Akt

## Slide 2
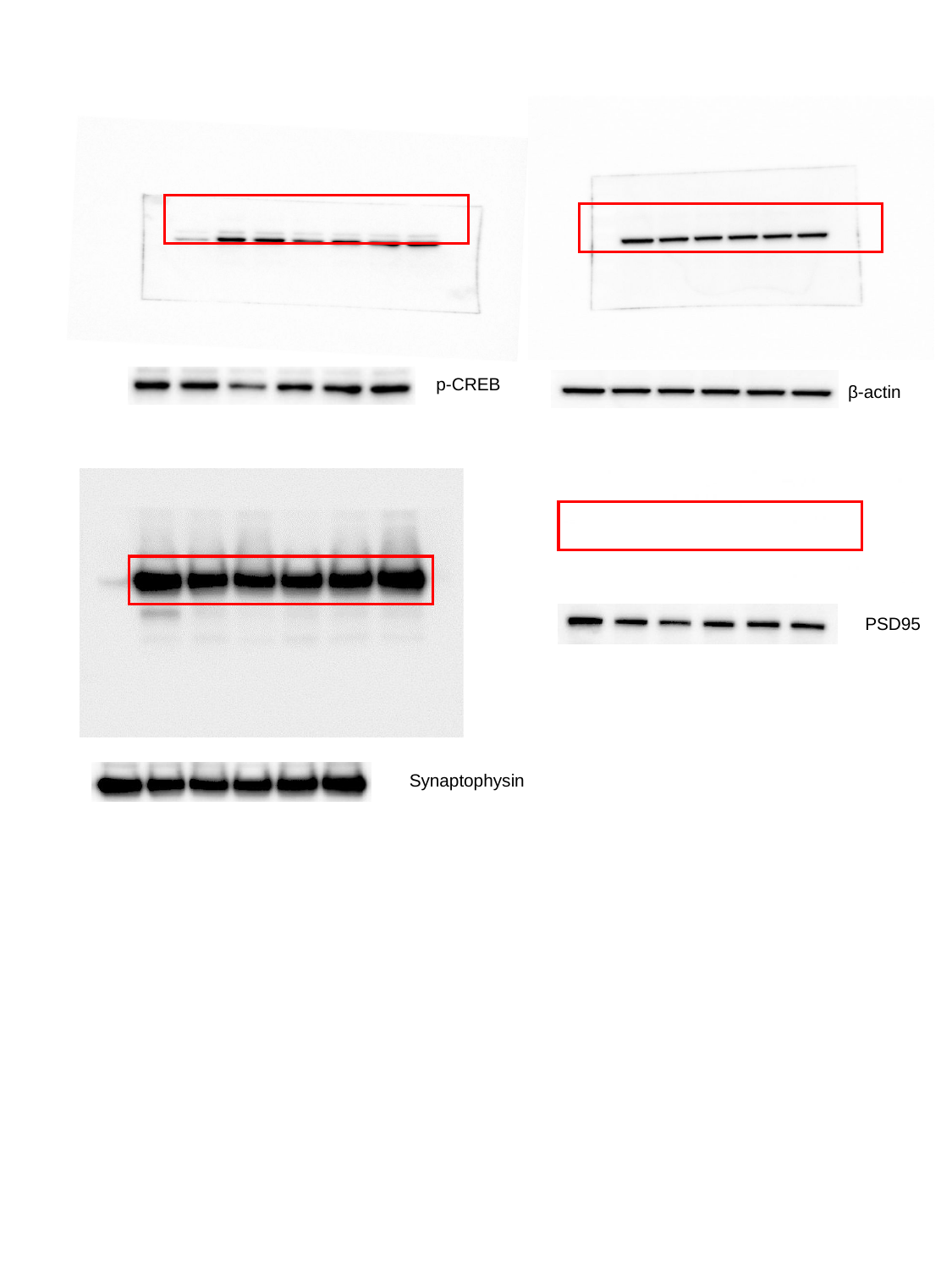

p-CREB
β-actin
PSD95
Synaptophysin
